# JNK Activation Correlates with Cognitive Impairment and Alteration of the Post-Synaptic Element in the 5xFAD AD Mouse Model

**DOI:** 10.3390/cells12060904

**Published:** 2023-03-15

**Authors:** Erica Cecilia Priori, Clara Alice Musi, Arianna Giani, Luca Colnaghi, Ivana Milic, Andrew Devitt, Tiziana Borsello, Mariaelena Repici

**Affiliations:** 1 Department of Pharmacological and Biomolecular Sciences, Università degli Studi di Milano, Via Balzaretti 9, 20133 Milano, Italy; 2 Mario Negri Institute for Pharmacolgical Research—IRCCS, Via Mario Negri 2, 20156 Milano, Italy; 3 Division of Neuroscience, IRCCS San Raffaele Scientific Institute, Via Olgettina 60, 20132 Milano, Italy; 4 School of Medicine, Vita-Salute San Raffaele University, Via Olgettina 58, 20132 Milano, Italy; 5 College of Health and Life Sciences, Aston University, Birmingham B4 7ET, UK

**Keywords:** c-Jun N-terminal kinase, synaptic dysfunction, cognitive decline, cell death

## Abstract

The c-Jun N-terminal kinases (JNKs) are a family of proteins that, once activated by stress stimuli, can alter neuronal functions and survival. The JNK cascade plays a crucial role in the post-synaptic neuronal compartment by altering its structural organization and leading, at worst, to an overall impairment of neuronal communication. Increasing evidence suggests that synaptic impairment is the first neurodegenerative event in Alzheimer’s disease (AD). To better elucidate this mechanism, we longitudinally studied 5xFAD mice at three selected time points representative of human AD symptom progression. We tested the mice cognitive performance by using the radial arm water maze (RAWM) in parallel with biochemical evaluations of post-synaptic enriched protein fraction and total cortical parenchyma. We found that 5xFAD mice presented a strong JNK activation at 3.5 months of age in the post-synaptic enriched protein fraction. This JNK activation correlates with a structural alteration of the post-synaptic density area and with memory impairment at this early stage of the disease that progressively declines to cause cell death. These findings pave the way for future studies on JNK as a key player in early neurodegeneration and as an important therapeutic target for the development of new compounds able to tackle synaptic impairment in the early phase of AD pathology.

## 1. Introduction

Alzheimer’s disease (AD) is a progressive and still untreatable neurodegenerative disorder. The last stage of the disease is the result of a pathological process already active and prolonged over time, characterized by synaptic dysfunction, synaptic loss, and eventually neuronal death. The c-Jun N-terminal kinase (JNK) mediates cellular stress and is deeply involved in AD pathogenesis [1,2,3]. Indeed, JNK phosphorylates both amyloid precursor protein (APP) and tau, promoting the production and accumulation of amyloid beta (Aβ) protein and neurofibrillary tangles deposition [4,5,6]. Interestingly, JNK plays also a role in regulating synapse physiology and formation [7,8,9] as well as dysfunction [3,10,11]. At the synaptic terminals JNKs are essential in synaptic plasticity [9], while prolonged overactivation of JNKs results in decreased protein levels in the post-synaptic density (PSD) region, triggering a mislocalization of glutamate receptors and eventually driving synaptic dysfunction [3,12]. The role of JNK in AD post-synaptic dysfunction was previously investigated in the CRND8 AD mouse model, where it was shown to regulate synaptic glutamate receptor trafficking by controlling phosphorylation of PSD95, eventually leading to neurotransmission impairments and spine loss [3,10,13]. Interestingly, JNK inhibition rescued both behavioral and biochemical degenerations in CRND8 mice [3,10,11], suggesting an essential role of JNK in AD-induced synaptopathy, dendritic spine loss, and neurodegeneration [14]. However, this mouse model does not mimic the long prodromal phase of plaque deposition, with the onset of the cognitive decline occurring at the same time as plaque deposition [15]. For this reason, the benchmark reference model in the AD field is currently the 5xFAD mice model [16].

The 5xFAD mouse overexpresses human APP and PSEN1, containing five familial AD mutations (APP KM670/671NL (Swedish), APP I716V (Florida), APP V717I (London), PSEN1 M146L, PSEN1 L286V) under the control of a Thy1-promoter. An in-depth analysis of these mice, performed by Forner and colleagues, confirmed that they develop amyloid pathology [16,17], microgliosis and inflammation [18,19,20], astrogliosis, impairments in long-term potentiation [21], and deficits in behavioral tasks [17,22]. In addition, differently from other AD models, cognitive decline appears months after amyloid plaque deposition [17]. Neuronal loss starts from 9 months of age in those areas involved in memory formation and processing, and 25–40% of layer 5 neurons are lost between 9 and 12 months [17,22,23]. This feature is particularly important as many other AD animal models do not present any neuronal loss. Interestingly, as in humans, synaptic dysfunction precedes neuronal death. On the other hand, spine and dendrite loss and axonal dystrophy start earlier, at around 4–6 months [17], when cognitive deficit usually begins. This suggests a stronger correlation between synaptic dysfunction and mild cognitive deficit. Thus, the 5xFAD mouse model enables the study of preventive treatments against AD synaptic dysfunction. Interestingly, JNK’s key role in 5xFAD was confirmed by chronic treatment with Brimaptide (also known as D-JNKI1), a peptide able to inhibit all JNK isoforms. This treatment resulted in a reduction of Aβ plaque burden, cognitive deficits, cell death, and the pro-inflammatory IL-1β cytokine [24,25]. Despite this experimental evidence, the relevance of JNK activation in the PSD of the post-synaptic element in this mouse model has never been explored. We here used behavioral, biochemical, and proteomic methods to characterize the sequence of events leading to JNK activation and cognitive decline in 5xFAD mice. We focused on three pivotal time points of the AD continuum: the pre-symptomatic phase (2 months), the initial phase (3.5 months), and the overt latest phase of the disease (10 months), deeply analyzing the molecular changes in cortical tissues.

## 2. Materials and Methods

### 2.1. Mice

Briefly, 5xFAD mice (B6SJL-Tg(APPSwFlLon,PSEN1*M146L*L286V) 6799Vas/Mmjax Strain #034840-JAX) on a B6SJLF1/J background were obtained from the Jackson Laboratory. Age-matched wild-type (wt) littermates were used as controls. Hemizygous male mice (not carriers for the Pde6brd1 gene) were bred to B6SJLF1/J female mice (carriers for the Pde6brd1 gene) at the Mario Negri Institute for Pharmacological Research IRCCS [3,26,27]. Procedures involving animals and their care were in accordance with national and international laws and policies (Permit Number 4/2021-PR). Genotyping was performed by PCR using a standard protocol provided by the Jackson Laboratory and GoTaq^®^G2 Flexi DNA polymerase kit (Promega, Madison, WI, USA). The experimental scheme comprised two groups: the Tg-5xFAD and the wt mice. Male and female tg-5xFAD and wt mice were tested at the ages of 2 (n = 7 per group), 3.5 (n = 7 per group), and 10 (n = 7 per group) months. 

### 2.2. Behavioral Test

The radial arm water maze (RAWM) was performed as described in Alamed et al., 2006 [28]. Entry into an incorrect arm (all four limbs within the arm) was scored as an error. If a mouse failed to make an arm entry within 15 s, this also was scored as an error. The errors for blocks of 3 consecutive trials were averaged for data analysis.

### 2.3. Triton Insoluble Fractionation (TIF)

After the behavioral test, animals were sacrificed, and brains were removed for biochemical analysis. Subcellular fractionation was performed as reported in the literature, with minor modifications [29,30].

### 2.4. Western Blot

Protein concentrations were quantified using the Bradford Assay (5000006, Bio-Rad Protein Assay, Hercules, CA, USA): 10 μg of total homogenate and 5 μg of TIF extracted proteins were separated by 10% SDS polyacrylamide gel electrophoresis. PVDF membranes (1620177, Bio-Rad, Hercules, CA, USA) were blocked in Tris-buffered saline 5% non-fat milk powder (70166, Sigma-Aldrich, Darmstadt, Germany), and 0.1% Tween 20 (P1379, Sigma-Aldrich, Darmstadt, Germany) (1 h, RT). Primary antibodies were diluted in the same buffer (incubation overnight, 4 °C) using anti-P-JNKs (1:1000, BK9251S, Cell Signalling, Danvers, MA, USA); anti-JNKs (1:1000 BK9252S, Cell Signalling, Danvers, MA, USA); anti p-APP (1:1000, Cell signaling); anti-APP (1:1000, VC298620, Invitrogen, Waltham, MA, USA); anti-p-c-Jun (1:1000, 06-659, Millipore, Bedford, MA, USA); anti-c-Jun (1:1000, BK9165S, Cell Signalling, Danvers, MA, USA); anti-NMDA Receptor 2A GluN2A (1:2000, BK4205S, Cell Signalling, Danvers, MA, USA); anti-NMDA Receptor 2B GluN2B (1:2000, BK14544S, Cell Signalling, Danvers, MA, USA); anti-Glutamate Receptor 1 (AMPA subtype) GluA1 (1:1000, BK13185S Millipore, Bedford, MA, USA); anti-Glutamate Receptor 2 (AMPA subtype) GluA2 (1:1000, MAB397 Millipore, Bedford, MA, USA); anti-post-synaptic density protein 95 (PSD95) (1:2000, CAY-10011435-100, Cayman Chemical Company, Ann Arbor, MI, USA); anti PSD95 (1:1000, AB2930, Abcam, Cambridge, UK); anti-Drebrin (1:1000, BSR-M05530, Boster, Pleasanton, CA, USA); anti-Shank3 (1:1000, 64555, Cell Signalling, Danvers, MA, USA); anti-Actin (1:5000, MAB1501, Millipore, Bedford, MA, USA). Blots were developed using horseradish-peroxidase-conjugated secondary antibodies (Santa Cruz Biotechnology, Dallas, TX, USA) and the ECL chemiluminescence system (Bio-Rad, Hercules, CA, USA). Western blots were quantified by densitometry using Quantity One software (Bio-Rad, Hercules, CA, USA). Experiments were performed at least three times.

### 2.5. ELISA

Aβ1-42 oligomers detection in cortex total homogenates was performed using the amyloid-beta (x-42) ELISA kit (IBL international GMBH, RE59721). Briefly, protein concentration was quantified using the Bradford assay and then diluted at the same concentration of 0.5 µg/µL (5000006, Bio-Rad Protein Assay, Hercules, CA, USA) following the manufacturer’s instruction. Data were shown as fold change of the average of all the wt groups.

### 2.6. Mass Spectrometry

Brain lysates (30 µg) were reduced in Laemmli buffer (15 min at 65 °C), then loaded and separated on 10% SDS-PAGE. Resolved proteins were stained with Coomassie Brilliant Blue G-250 (0.5% *w*/*v* in 40% aqueous methanol and 10% glacial acetic acid) overnight at 4 °C. Gels were de-stained and all samples were divided into five bands between exact molecular weight markers and across the whole gel, providing five gel sections for every sample. Excised sections were separately diced and transferred into PCR-clean low protein binding polypropylene tubes (Eppendorf, Hamburg, Germany). Gel pieces were fully de-stained with 50% acetonitrile in 50 mM ammonium bicarbonate (50% MeCN *v*/*v* in 50 mM ABC), then dehydrated with pure acetonitrile and vacuum dried for 30 min in a vacuum concentrator (Eppendorf, Hamburg, Germany). For proteolytic in-gel digestion, dried gel pieces were fully rehydrated with 40 µL of trypsin (Sequencing grade, Promega, UK) in 6 mM ABC (25:1 protein to trypsin ratio) at RT for 5 min and layered with 200 µL of 6 mM ammonium bicarbonate to prevent the gel from dehydrating during overnight digestion at 37 °C on an orbital thermoshaker (700× *g*). Tryptic peptides were extracted from the gel sequentially in 30% and 50% of acetonitrile in 50 mM ABC (15 min in an ultrasonic bath), after which samples were fully dehydrated in pure acetonitrile. Peptide extracts from a single-sample section were combined into one polypropylene tube, after which they were vacuum dried and stored at −20 °C before mass spectrometry analysis.

Before analysis, dried samples were reconstituted in 30 µL of 3% aqueous acetonitrile and 0.1% formic acid for liquid-chromatography-coupled tandem mass spectrometry (LC-MS/MS) analysis. Peptide samples (5 µL) were injected onto a trap column (nanoEase M/Z Symmetry C18 Trap Column, 100Å, 5 µm, 180 µm × 20 mm, Waters, UK) on an nUPLC system (Acquity M class, Waters, UK) operating in a single-pump trapping mode at the flow rate of 5 µL/min of eluent B (eluent B: acetonitrile in aqueous 0.1% formic acid). Peptide separation was achieved at a flow rate of 0.5 µL/min on an analytical column (AcclaimTM, PepMapTM C18, 3 µm, 100 Å, 75 µm × 150 mm, ThermoScientific, UK) using 1% of eluent B (acetonitrile in aqueous 0.1% formic acid) applying the following gradient: 0–45 min 1–45% B, 45–49 min 45–90% B, 49–52 min 90% B, 52–67 min 1% B. Stable peptide electrospray was formed at 2200 V using a PicoTipTM emitter (New Objective, Germany), and charged peptides were analyzed on 5600 TripleTof mass spectrometer (AB Sciex, Framingham, MA, USA) in information-dependent mode selecting the 10 most intense ions from each high-resolution MS survey scan for high sensitivity MS/MS. Acquired peptide ions were temporarily excluded from MS/MS acquisition for 30 s. The mass spectrometer was calibrated prior to acquisition to ensure a high mass accuracy on both MS and tandem mass spectrometry (MS/MS) levels.

Relative protein quantification was performed using Progenesis QI for proteomics software (version 4, Nonlinear Dynamics, Newcastle, UK). Each sample sub-group (within the same molecular weight range) was aligned on a retention time vs. *m*/*z* plot, ensuring the alignment of identical peptides across different samples. Data was further in-silico normalized (based on the peptide distribution) to allow for the high-accuracy relative quantification. All sub-groups were combined using a multi-fraction setup to build up representative samples. Relative quantification included only protein-unique peptides. Merged data were exported as an .mgf file to Mascot search engine (Mascot Daemon platform, ver 2.5) which was searched against the curated SwissProt database with the following parameters: mass tolerance of 0.1 Da for MS and 0.5 Da for MS/MS spectra, a maximum of two trypsin missed-cleavages, Mus Musculus taxonomy, and variable modifications of methionine oxidation and cysteine carbamidomethylation. Mascot searches were exported back to Progenesis for the protein relative quantification between the samples. All keratins were treated as contamination and were excluded from the analysis. Exported protein relative quantification data tables were used for the quantitative gene ontology analysis using FunRich software tool.

### 2.7. Statistical Analysis

Statistical analyses were performed with the Graph Pad Prism 9 program. Data were analyzed with Student’s *t*-test and expressed as mean ± SEM with statistical significance *p* < 0.05. For comparison between multiple groups, two-way ANOVA was used, followed by Tukey’s post hoc test.

## 3. Results

We analyzed 5xFAD at three different time points representing: (1) the pre-symptomatic phase (2 months of age); (2) the early phase (3.5 months of age); and (3) the late phase of the disease (10 months of age).

### 3.1. Pre-Symptomatic Phase: 2-Month-Old 5xFAD Mice Showed an Increase in APP and P-APP Levels but No Cognitive Deficit or JNK Activation

**Figure 1:**
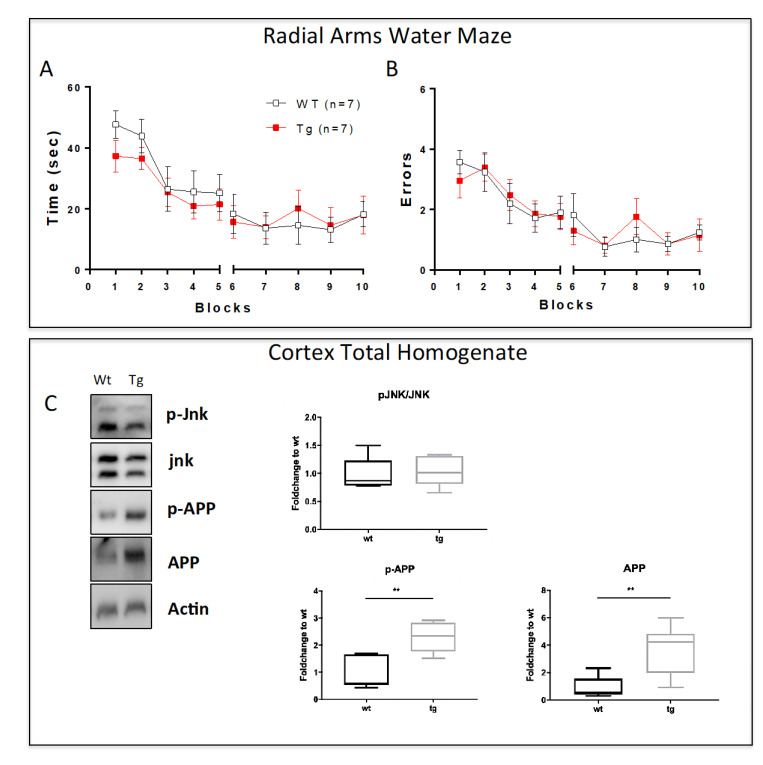
Absence of memory impairment and JNK activation in 2-month-old 5xFAD mice. (**A**) Time and (**B**) number of errors to find the submerged escape platform demonstrate the absence of cognitive impairment in 2-month-old 5xFAD mice. (**C**) Representative Western blots and relative quantifications of cortical total homogenate show higher levels of p-APP, APP, and no differences in p-JNK/JNK ratio in 5xFAD vs. wt mice. Genotypes are compared using *t*-tests. Statistical significance: ** *p* < 0.01. Data are expressed as mean ± SEM.

5xFAD mice cognitive deficits were investigated using the RAWM to evaluate the number of errors and the time taken to locate the escape platform. As expected, 2-month-old 5xFAD mice did not show memory impairment compared to wt animals; no significant differences were found between the two groups in all the trials (Figure 1A,B), confirming a pre-symptomatic phase.

On the same animals, Western blot analysis was performed on cortical total homogenate. JNK activation was evaluated by measuring the ratio between the phosphorylated (p-JNK) and the total form of the kinases (JNK), and no differences were found in tg compared to wt animals (Figure 1C). In addition, we also analyzed total P-APP and APP levels using an antibody that recognizes the specific JNK phosphorylation site on this protein (T669). Our results show that despite the absence of cognitive deficit at 2 months, a significant increase in both p-APP and APP protein levels (*p* < 0.01) was observed in tg-5xFAD mice compared to wt animals (Figure 1C), highlighting the early accumulation of amyloid proteins [17].

### 3.2. Early Symptomatic Phase: Cognitive Impairment Correlates with Increased JNK Activation, Aβ Oligomers Deposition, and Synaptic Dysfunction in 3.5-Month-Old 5xFAD Mice

**Figure 2:**
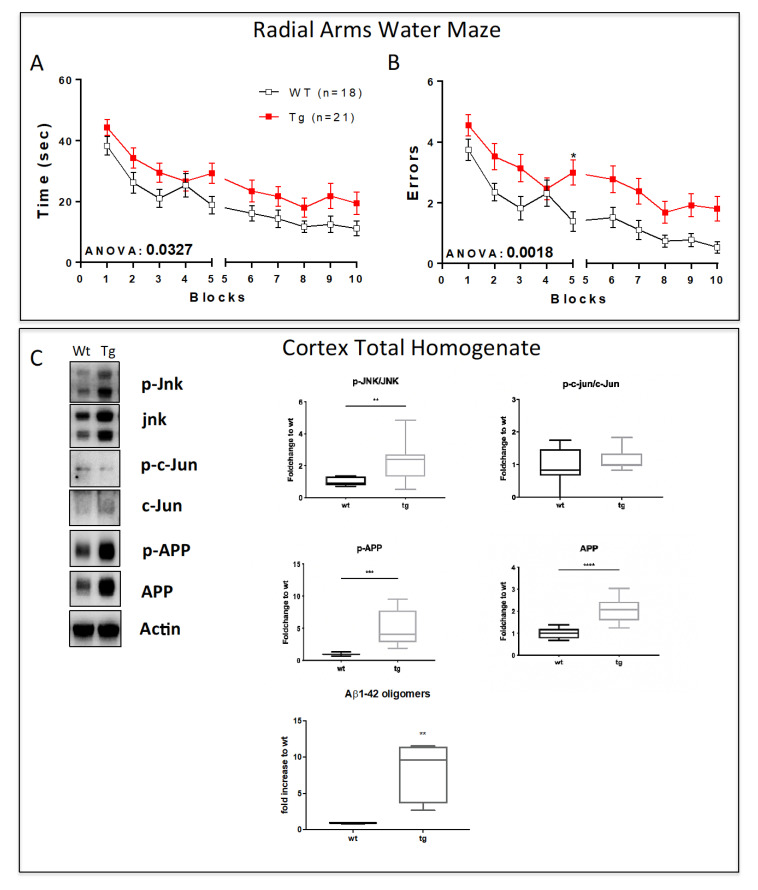
Activation of JNK signaling pathway correlates with cognitive impairment in 3.5-month-old 5xFAD mice. (**A**) Time and (**B**) number of errors to find the submerged escape platform demonstrate an initial cognitive impairment in 3.5-month-old tg-5xFAD mice. (**C**) Representative Western blots and relative quantifications of cortical total homogenate show higher levels of p-JNK/JNK ratio, p-APP, and APP but no statistical differences in p-c-Jun/c-Jun ratio in 5xFAD vs. wt mice. Elisa analysis shows an 8.6 fold increase in Aβ1-42 oligomers in cortical total homogenate in 5xFAD mice. Genotypes are compared using *t*-tests. Statistical significance: * *p* < 0.05, ** *p* < 0.01, *** *p* < 0.001, **** *p* < 0.0001. Data are expressed as mean ± SEM.

We next analyzed mice at 3.5 months of age. Wt and 5xFAD mice performed differently in the RAWM and we found a significant effect of the genotype on both the errors and the time spent performing the behavioral test (ANOVA for time, *p* = 0.032, and for errors, *p* = 0.0018; Figure 2A). These data suggest an initial phase of cognitive impairment in 5xFAD mice at 3.5 months of age. Interestingly, the biochemical analysis showed a statistically significant increase in JNK activation (Figure 2B, *p* < 0.01) with no significant differences in the phosphorylation of its elective target c-Jun [31,32,32] (Figure 2C), which highlights an activation of the JNK signaling pathway with no involvement of neuronal death. Additionally, 3.5-month-old transgenic mice also displayed increased APP levels compared to wt mice (Figure 2B, *p* < 0.001) and increased p-APP levels (Figure 2B, *p* < 0.0001) as expected. We then performed an ELISA assay on cortical total homogenate to evaluate the deposition in the parenchyma of Aβ1-42 oligomers, the most toxic species deriving from APP processing [33]. Our results showed a clear increase in Aβ1-42 oligomers in tg-5xFAD mice compared to wt (*p* < 0.01, Figure 2C).

**Figure 3:**
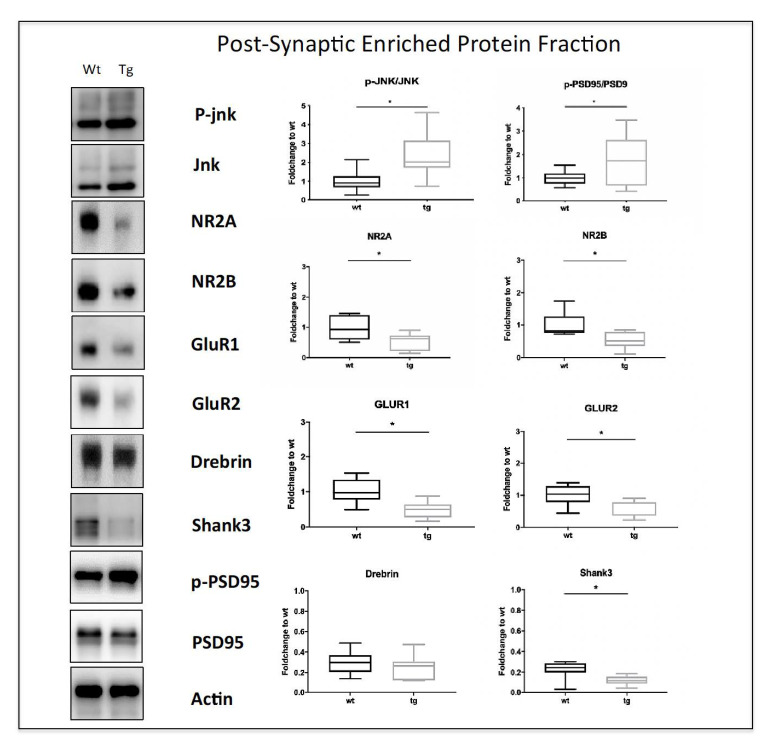
Synaptic dysfunction in the cortical post-synaptic proteins enriched fraction of 3.5-month-old mice. Representative Western blots and relative quantifications of 3.5-month-old mice TIF cortex show JNK signaling activation in tg compared to wt mice and increased levels of p-PSD95/PSD95 ratio together with decreased levels of NR2A, NR2B, GluR1, GluR2, and Shank3 levels. Genotypes are compared using *t*-tests. Statistical significance: * *p* < 0.05. Data are expressed as mean ± SEM.

The cognitive impairment observed at 3.5 months in 5xFAD-tg mice prompted us to study the synaptic dysfunction in these mice. We then performed a biochemical characterization of the cortical post-synaptic enriched protein fraction (Triton Insoluble Fraction-TIF) of wt and transgenic mice representing the PSD-region. Interestingly, the p-JNK/JNK ratio was increased in the TIF fraction of 5xFAD mice (Figure 3 *p* < 0.05), confirming a strong JNK signaling activation in the PSD region. Additionally, our data showed a dysregulation of PSD95, the main scaffold protein of the excitatory synapses. PSD95 is also one of the JNK targets in the TIF compartment [7,34], and we found an increase in P-PSD95/PSD95 ratio (Figure 3, *p* < 0.05) in tg-5xFAD vs. wt mice. In contrast, the levels of Shank3, another scaffold protein, and JNK target of the excitatory post-synaptic terminal [35], were decreased in transgenic compared to wt mice (*p* < 0.05 Figure 3), while Drebrin, a marker of mature spines, levels did not change between tg and wt mice. We also evaluated glutamate receptor levels by looking at NR2A and NR2B as representatives of NMDA receptor subunits, and GluR1 and GluR2 as representatives of AMPA receptors. We observed a statistically significant decrease in all the subunits evaluated in tg-5xFAD mice compared to wt (*p* < 0.05, Figure 3), indicating a strong disruption of the PSD biochemical organization.

### 3.3. The Late Phase of the Disease: 10-Month-Old 5xFAD Mice Cortical Lysates Showed Strong Activation of the Cell Death Programs

**Figure 4:**
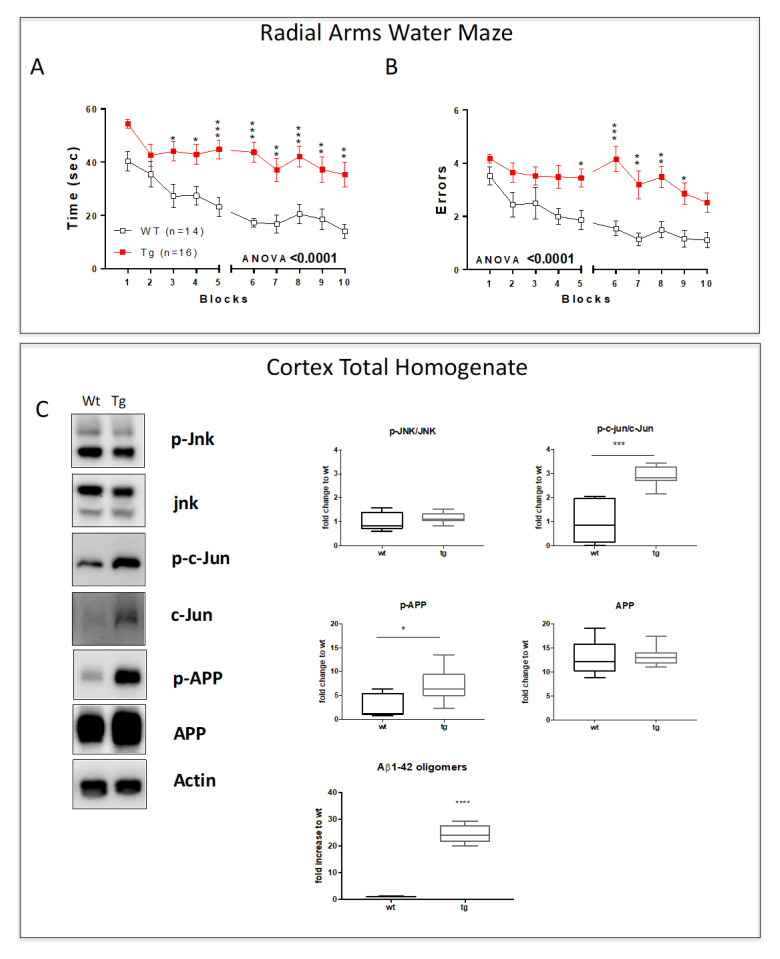
Cognitive impairment and parenchyma degeneration in the cortex of 10-month-old mice. (**A**) Time and (**B**) number of errors to find the submerged escape platform demonstrate the strong cognitive impairment in tg-5xFAD mice compared to wt. (**C**) Representative Western blots and relative quantifications of 10-month-old mice cortical total homogenate show a higher level of p-c-Jun/c-Jun ratio and P-APP in tg-5xFAD vs. wt mice and no changes in p-JNK/JNK and APP levels. Elisa Aβ1-42 oligomers evaluation in the cortical total homogenate. Genotypes are compared using *t*-tests. Statistical significance: * *p* < 0.05, ** *p* < 0.01, *** *p* < 0.001, **** *p* < 0.0001. Data are expressed as mean ± SEM.

We next analyzed mice at 10 months of age, and 5xFAD mice failed to learn the platform location over 2 days of testing, displaying an almost flat learning curve, and were significantly impaired compared to the non-transgenic mice (ANOVA for time, *p* < 0.0001, and for errors, *p* < 0.0001; Figure 4A,B). These results demonstrated an important worsening of cognitive and mnemonic abilities in 5xFAD animals compared to wt mice, confirming this as the latest phase of the pathology. When we looked at the cortex lysates, no JNK activation was found in tg compared to wt mice, while the p-c-Jun/c-Jun ratio was sharply increased (*p* < 0.001, Figure 4C), indicating the activation of the neuronal death program. Our data also showed increased p-APP levels (*p* < 0.05, Figure 4C) without any differences in APP expression in 5xFAD vs. wt mice. However, we observed sharply increased Aβ1-42 levels (*p* < 0.0001, Figure 4C) in 5xFAD mice, reinforcing the correlation between the progression of the disease and the cognitive impairments.

**Figure 5:**
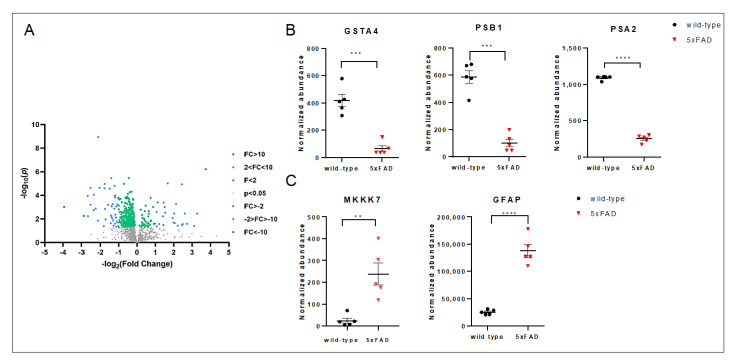
Mass spectrometry analysis of cortex lysates of 5xFAD and wt mice at 10 months of age. (**A**) Volcano plot illustrates significantly differentially abundant proteins. The −log10 (Benjamini–Hochberg corrected *p* value) is plotted against the log2 (fold change: 5xFAD/wt). *p* = 0.05 is the significance threshold (prior to logarithmic transformation). (**B**) Significant decrease was observed in GSTA4, PSB1, PSA2 proteins in tg vs. control mice. (**C**) A significant increase in 10-month-old 5xFAD lysates was observed for GFAP and MKKK7 proteins. Genotypes are compared using *t*-tests. Statistical significance: ** *p* < 0.01, *** *p* < 0.001, **** *p* < 0.0001. Data are expressed as mean ± SEM.

To gain insight into the role of JNK in processes impaired by such a high increase in Aβ oligomers we looked at the proteomic data in the cortex of 5xFAD mice compared to wt. Our results, as expected, showed a very different proteomic signature of 5xFAD mice compared to wt (Figure 5). We screened for differentially expressed proteins between wt and 5xFAD mice via group *t*-test and identified 389 differentially expressed proteins. Volcano plot, indicating the fold change in expression and the statistical significance for 5xFAD versus wt, is shown in Figure 5A. We next used these results to identify protein candidates whose role in neuronal death could be linked to the strong c-jun activation observed at 10 months. We found a 6.4-fold decrease in the antioxidant and detoxifying enzyme GST4A (*p* < 0.001) and a 5.8- and 4.33-fold reduction in proteasome subunits PSMB1 and PSMA2, respectively (*p* < 0.001 and *p* < 0.0001, Figure 5). We also found a 5.45-fold increase in GFAP and a 9.7-fold increase in MKKK7, whose activation induces the apoptotic pathway and which is an upstream activator of JNK (*p* < 0.0001 and *p* < 0.01, Figure 5), confirming JNK as a key player in the cell death program in 10-month-old 5xFAD tg mice.

## 4. Discussion

The first indication of a role of JNK in AD pathology was its activation markedly increased in AD human brains, where activated JNK was found closely associated with neurofibrillary tangles, senile plaques, neuropil threads, and granulovascular degeneration structures [36]. Further studies showed that JNK phosphorylates both amyloid precursor protein, promoting the APP cleavage in Aβ-amyloid oligomers, and tau, furthering the deposition of neurofibrillary tangles [37,38]. In addition, the JNK3 level was found to increase in the CSF of a small cohort of AD patients and correlated with the extent of cognitive impairment [39]. Beyond its direct action on APP and tau, JNK phosphorylates components of the synaptic machinery and is involved in synaptic plasticity changes [7,9]. Indeed, JNK is strongly involved in the phosphorylation of the tSNARE proteins, thus regulating the zipping of the neurotransmitter vesicles in the pre-synaptic element [37]. It also phosphorylates PSD95 and SHANK3, the most abundant scaffold proteins of the PSD region [12,40]. In addition, JNK participates in the inflammatory reaction and the neurodegeneration program [3,10,25]. In AD, synaptic changes occur in the early stage of the disease before Aβ plaque formation and neuronal loss [41], but the molecular mechanisms responsible for synapse dysfunction and loss remain poorly understood. However, this early symptomatic stage of pathology can represent a therapeutic window to treat AD and other diseases. The 5xFAD mouse model is the benchmarked reference model in mimicking human AD pathology and represents a viable model for preclinical testing [16]. We here analyzed biochemical alterations in 5xFAD mice brain parenchyma from 2 to 10 months of age, focusing on a specific postsynaptic element as a neuronal subcellular compartment to study synaptic dysfunction.

The two-month-old 5xFAD mice showed an increase in p-APP in cortex total homogenates without JNK activation and cognitive decline. This stage, without any kind of lesion or alteration of intracellular pathways, is defined as a pre-symptomatic phase in the 5xFAD mouse model. At 10 months of age, we observed a stronger increase in P-APP and Aβ 1-42 oligomer levels that correlated with robust memory impairment and ongoing cell death characterized by a strong increase in p-c-jun, the elective JNK target. This indicates an ongoing cell-death pathway in neurons. JNK has many apoptotic targets and regulates the activity of transcriptional factors in the nucleus (c-Jun, ATF2, and c-Fos), but it also phosphorylates proapoptotic BH3-only proteins [42] and regulates the balance between anti-apoptotic Bcl-2 and pro-apoptotic Bax proteins [43]. We next decided to use proteomic analysis to deeper characterize this late pathological phase with cognitive decline, focusing on the relation with JNK signaling. We detected a significant decrease in GSTA4 in 5xFAD compared to wt mice. Decreased glutathione levels were observed in mouse models of Alzheimer’s disease [44] and in AD patients [45]. It is well known that oxidative stress, caused by an imbalance in free radical production and cellular antioxidant response, plays a key role in the pathogenesis of AD. JNK plays a pivotal role in oxidative stress-activated cellular response, which contributes to cell death. Recent work has also shown that in neurons, JNK is activated specifically by oxidative stress and activates c-jun containing AP-1 transcription factors independently of c-Fos. This specific c-jun activation results in an increased abundance of the antioxidant protein SRXN-1 [46]. Thus, the strong activation of c-jun we detected in 5xFAD lysates at 10 months could represent an attempt to counteract the decrease in GST. Accumulation of Aβ aggregates has been shown to inhibit proteasome activity, thus contributing to the increase in oxidative stress which in turn triggers c-jun activation [47]. Interestingly, we observed a strong decrease in proteasome subunit beta type 1 and alpha type 2 in 5xFAD mice versus control. Lastly, our proteomic analysis showed a strong increase in GFAP and MKKK7 protein levels in the cortex of 5xFAD mice compared to controls. While the increase in GFAP protein in the cortex and hippocampus in AD mouse models is well known [48,49], a consensus AP-1 binding site is present in the human GFAP promoter and is conserved in mice and rats. Interestingly, this AP-1 site was shown to be essential for the upregulation of a gfa2-nlac transgene in response to injury [50,51]. This could indicate a role for JNK/c-jun activation at 10 months in GFAP overexpression in the late phase of the disease. The 9.68-fold change in MKKK7 expression in 5xFAD mice strengthens the role of both MKK/JNK and p38 MAPK signal transduction cascade and correlates with the increase in c-jun shown in our Western blot analysis at 10 months of age. Taken together, our proteomic data show that the strong cognitive decline at 10 months of age correlates with an ongoing neuronal death process highlighted by strong activation of the JNK signaling pathway. It is clear that the time point of 10 months is too late for any therapeutic intervention.

**Figure 6:**
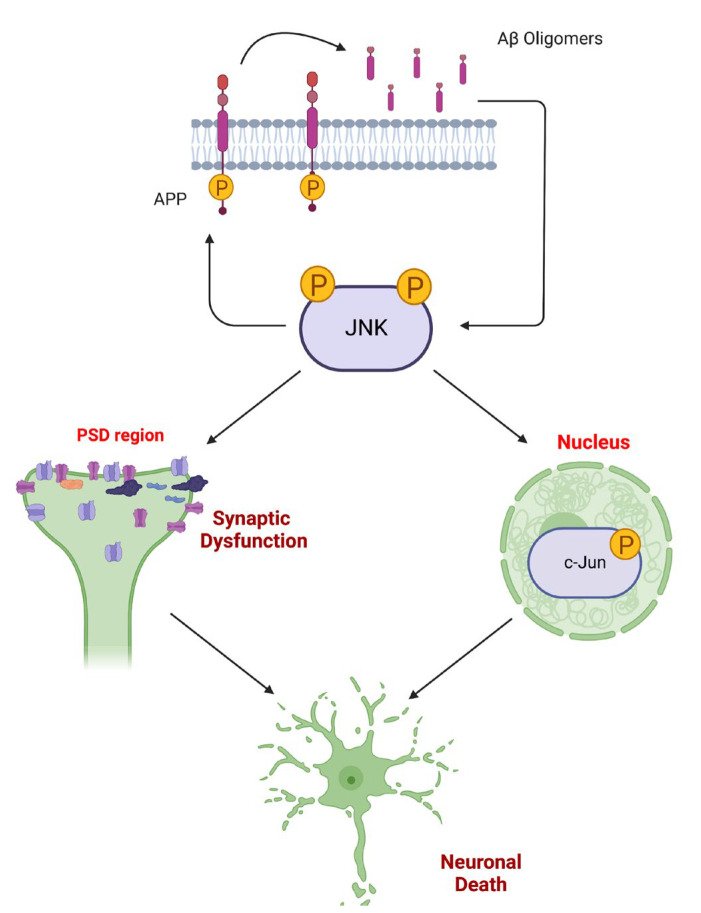
JNK role in AD pathogenesis. Stress stimuli (such as Aβ oligomers) induce JNK hyper-activation that acts in distinct cell compartments. Active JNK phosphorylates APP, promoting its amyloidogenic cleavage and the formation of Aβ oligomers that, in turn, activate JNK. While AD synaptic dysfunction can be induced directly by Aβ oligomers by promoting the misfunctioning of glutamate receptors, JNK plays a role in the post-synaptic element by phosphorylating its targets (i.e., PSD95), perpetrating the neurodegenerative process. Additionally, activated JNK can also translocate in the nucleus, where it targets the elective substrate c-jun, thus triggering the neuronal death pathway.

To gain insight into the link between earlier events in AD pathogenesis and the JNK pathway, we next evaluated 3.5 months as a time point representative of an early symptomatic phase with the beginning of a mild cognitive decline. Strikingly, we found a strong JNK activation in both the cortex lysate as well as in the post-synaptic enriched protein fraction. Together with this strong JNK activation, we detected a strong decrease in the main excitatory post-synaptic density biomarkers (glutamate receptors-NR2A, NR2B, GluR1, GluR2) and the scaffold proteins (PSD95, Shank3). This strongly suggests that JNK activation mirrors the alteration of the postsynaptic element at this early phase of the disease. We previously demonstrated in the CRND8 AD mouse model that Aβ oligomers powerfully trigger JNK activation before PSD alteration [3,10,11,13], and we proved the key role of JNK by using D-JNKI1, a cell-permeable peptide that inhibits all JNK isoforms. JNK inhibition by D-JNKI1 was able to prevent synaptic impairment and behavioral deficit. In agreement with these data, we here show that JNK activation at 3.5 months of age in the 5xFAD mouse model correlates with mild cognitive impairment and an alteration of the post-synaptic element, thus confirming what was observed before. Notably, at 3.5 months of age, we did not observe any c-jun activation, thus confirming that JNK, during this early phase, is not yet acting on targets involved in the neuronal death program (Figure 6).

## 5. Conclusions

In summary, the present study indicates a different role of JNK in the early symptomatic phase compared to a later stage of AD pathogenesis in 5xFAD mice. While JNK activation at 3.5 months of age, when behavioral abnormalities become detectable, correlates with postsynaptic dysfunction, c-jun activation at 10 months of age correlates with neuronal death. These data strongly show that JNK is early activated in the pre-symptomatic AD-phase, and its activation persists up to the late phase of the disease, suggesting a key role for JNK in AD and its potential as a therapeutic target. More importantly, we here propose JNK as an ideal target to block PSD disruption in the early AD phase, paving the way for future studies on specific JNK inhibition as a tool for blocking synaptic dysfunction in AD.

## Data Availability

Data are available upon reasonable request to the corresponding author.
